# Abstracts and Gleanings

**Published:** 1880-04-20

**Authors:** 


### ABSTRACTS AND GLEANINGS.

**Treatment of Yellow Fever.**—Dr. W. L. Coleman, of Texas, says in the New Orleans Medical and Surgical Journal:

While I regard calomel and quinine as poison to a yellow fever patient, many distinguished gentlemen in the profession depend upon them as their sheet-anchor; but I am thoroughly convinced that we have discovered no antidote or abortive treatment for the disease, and, since I have abandoned the use of powerful remedies, have had far better success with the expectant method. I do not, however, intend to enter on a dissertation upon the treatment, but will content myself by mentioning a few of what I consider the most important points in the management of a yellow fever case.

1. Move the bowels as early as possible in the disease, with a mild laxative or an enema of tepid water, and then let them remain quiet until the fever has run its course.

2. Keep the patient perfectly quiet, in a horizontal position, and let him see no one but his physician and nurse.

3. Avoid giving anything that will offend the stomach; and, if there is nausea, try to relieve it by the free use of rubefacients, and, if necessary, by a blister.

4. Keep the feet warm and the head cool, by applying bottles of hot water to the feet, and iced *eau sedative* freely to the head.

5. Allow the patient as much ice as he desires, and use diluents freely, consisting of warm teas, flax-seed water, lemonade, hot or cold, to suit the taste, and keep a strict supervision over the condition of the kidneys. While medicine is needed in a great many cases, yet I feel confident too much was given in the past epidemic.

The treatment at the Louisville Hospital, as given by Dr. J. B. Marvin, resident physician, in the American Practitioner, was in part as follows:

For nervousness and sleeplessness, chloral hydrate is the best remedy; give thirty grains each of chloral hydrate and bromide of potassium, in one or two ounces of warm milk, by enema. This combination has a most happy sedative effect; it also lowers the temperature. In some cases, tincture of hyoscyamus has a happy effect.

Patients are very sensitive to the effects of opium and its salts; very small doses, either by the mouth or rectum, producing alarming narcosis. Opium did not appear to interfere with the proper functions of the kidneys; its use was discontinued solely on account of its effects on the brain. A number of patients were given morphia while *en route* to this city; they never came from under the influence of the drug, but lay in a stupid, lethargic condition, pupils contracted, and died with all the appearances of narcotic poisoning. In some patients, who were constant eaters of morphia, most distressing appeals were made for it. Various devices were resorted to in order to deceive them. About a fourth of a grain of quinia in powder was given, and followed by thirty grains of chloral hydrate. If this failed, a hypodermic injection of eight or ten minims of water had the desired effect, quieting the cries of the patient, and putting him to sleep.

If hemorrhage from the nose or mouth became free, a spray of Monsel's solution (half strength) always checked it. For hemorrhages from the stomach and bowels, if deemed advisable to check them, ten minims of Monsel's solution were given every hour. For renal hemorrhage, ten grains of gallic acid were given every three hours. In some cases fifteen-minim doses of aromatic sulphuric acid were substituted.

Stimulants are required in every case. Port wine proved most acceptable and beneficial, it being retained when the stomach rejected everything else. Acid wines and *champagnes* disagreed in every case; complaints of their bad effects were so general that they were discontinued. Brandy and whiskey were given in all cases, either by mouth or rectum. During convalescence, ale and beer were much relished, and were freely given. During the first two or three days of the attack, the less food taken the better. Milk, chicken broth, beef essence, etc., according to the desire of the patient, were given in small quantities every hour. During convalescence, oyster soup, soft-boiled eggs, crackers, toast, etc., in moderate quantity are given. Great care is required to prevent the patient from over-eating. Not a single relapse occurred in this hospital, which is largely attributable to the great caution exercised in regulating the dietary of the patients.—*Half-Year Comp. of Med. Science.*

**Naso-Pharyngeal Catarrh.**—A clinical lecture of J. Solis Cohen, M.D., Lecturer on Diseases of the Throat, etc., Jefferson Medical College, Philadelphia, is published in the Medical News and Library, and it is believed that the views of one of the best American authorities on the treatment of this frequent, obstinate, and most discouraging affection, will prove acceptable to the readers of this department.

Naso-pharyngeal Catarrh is an inflammation of the mucous membrane of the nasal cavities and upper part of the pharynx. It produces a tenacious secretion of mucus, which blocks up the nasal fossæ and tends especially to collect in the roof of the pharynx, just behind the nasal septum, where the glands are abundant. This secretion is removed partly by blowing the nose and partly by hawking it down into the pharynx and then expectorating it. Occasionally scabs and crusts are expelled. The secretion, being retained, undergoes decomposition, leading to the evolution of fœtid gases and consequent foul breath. The disease may extend to the frontal and maxillary sinuses, producing brow and face-ache; it may also cause closure of the apertures by which the nasal fossæ communicate with these sinuses, and consequent abscess, cystic tumor, or morbid growth in the latter.

The most important element in the treatment is thorough removal of the accumulated mucus. This should be done daily, and is often alone sufficient for the cure of simple inflammatory cases. The retained secretion and the decomposed gases irritate the diseased membrane still further, thus keeping up and intensifying the morbid condition; moreover, breathing the foul air impairs the general health and even sometimes leads to slow septic poisoning.

For removal of the discharge, a solution of salt in tepid water (ʒj to Oij) is usually employed; in mild cases this may be snuffed into the pharynx through the nasal cavities very effectively; otherwise it may be applied by means of the syringe, spray-apparatus, or Thudicum's nasal douche.

In using the douche, the mouth should be open, and the patient cautioned not to swallow, lest the fluid be forced through the eustachian tubes and produce otitis media; if the fluid be warm, however, there will be but little danger, even should such an event occur. About 1qt. of the solution should be used once or twice a day. The fluid may also be injected from behind by means of a curved syringe.

Frequently applications have to be made to the posterior portion of the nasal passages; this may be done by means of a rectangular probe, firmly attached to the end of which is a small piece of sponge saturated with the medicament (as, for instance, equal parts of glycerite of tannin and compound solution of iodine). For this operation the mouth should be well illuminated, and tongue depressed with a spatula. The sponge should be forced into first one posterior nasal outlet and then, after waiting a few minutes, into the other. This application is to be repeated three times a week. Another method of local treatment, in which a medicated solution is retained in contact with the parts for from 20 to 30 minutes, is by flexible bougies made of gelatine impregnated with the remedy (as gr. ij of sulphate of zinc and gr. ss of carbolic acid). The bougie gradually dissolves in the nasal cavity. To prevent its dropping into the throat, a string is passed through it, which is attached to the patient's ear.

Ulcers are rare in simple inflammatory catarrhs, but frequent and often extensive and deep in tuberculous, scrofulous and syphilitic subjects.

After cleansing the nasal passages, their interior may be examined, before a good light, by drawing the wing of the nostril aside, with a hair-pin bent into the form of a hook, which is as efficient as any nasal speculum.

In constitutional diathesis, appropriate constitutional treatment is necessary, and, of course, the removal of dead bone, foreign bodies, etc., is a sine qua non of cure.—*Md. Med. Journal.*

**Surgical Treatment of Pleuritic Effusions.**—In an article in the Canada Lancet, Dr. A. McKay writes of the treatment of pleuritic effusions :

The drainage tube and local antiseptic treatment seems to be gaining ground, and I think we are indebted to a Canadian, Dr. Richardson, of Toronto, for its first introduction into practice in Canada. His case treated in 1869 is, at all events, the first recorded here, and I am glad to say that it proved successful. According to a number of writers on the subject, the great danger to be apprehended, is the admission of air into the cavity; but if you will consider for a moment the form of the chest, with a non-yielding external wall, and also the probability of adhesions, surrounding the contracted lung, more especially in cases of long standing, it would not only be unscientific, but positively injurious, to attempt the withdrawal of fluid, and at the same time prevent the entrance of air into the cavity. It is well known that after air is admitted, that it changes the nature of the pus, and it sometimes very rapidly becomes offensive. This change would be a serious objection, providing it would increase the liability to absorption, but we have every proof to the contrary. The exclusion of air is also recommended on the supposition that it will interfere with the expansion of the lung; but we know that atmospheric pressure is the same, whether internal or external to the wall of the chest, and it could not possibly offer any resistance to the expanding lung, unless the opening could be hermetically sealed, which, under the circumstances, would be a very difficult undertaking. Again, if we attempt the exclusion of air for the purpose of facilitating the lung expansion, its place must either be supplied by fluid, or the expanding lung itself; but the attempt to rapidly expand the lung by means of a vacuum, might endanger the patient's life by forcible laceration of the adhesions or pleura.

In cases of this kind the aspirator should never be used, under any circumstances, for the following reasons :

1st. It will not remove all the fluid in cases of long standing.

2d. It will not prevent re-secretion of fluid.

3d. Its employment is attended with danger in recent cases, from the point of the needle coming in contact with the expanding lung.

4th. Where the fluid is purulent the operation must be repeated, causing more inconvenience to the patient, besides the danger of piercing the lung, and in that way complicating the disease.

5. The main object to be obtained by its use, viz., the exclusion of air from the cavity, is not now considered necessary, for it is admitted on all hands, that the admixture of air with serous fluid, will not lead to its becoming purulent.—*Half-Yearly Compendium of Med. Science.*

**Mysophobia.**—Mysophobia is a species of mental derangement attended by a morbid fear of defilement or contamination. The following case, from the Indiana Practitioner, reported to N. Y. Neurological Society, by Dr. W. A. Hammond, will serve to illustrate this singular affection.

Miss F——, aged eighteen, tall and slender, consulted me, January 23d, of the present year. From herself and her mother I obtained the following history :

About eighteen months previously she had gone to stay in the country with some friends, and on one occasion slept in a farm-house. On her return home she at once took a bath, and had her head, the hair of which was very long and thick, thoroughly washed. To her great surprise and disgust, it was found to be full of lice. She had always been exceedingly cleanly as regarded her person, and the shock she experienced on learning of the presence of these parasites completely unnerved her. She insisted on repeated washings of the head with soap, carbolic acid and other detergent and disinfectant substances, and even then was not convinced that all the vermin had been destroyed.

This was the starting point of all the subsequent mental disturbance. Little by little the idea became rooted that she could not escape sources of contamination, that other persons might defile her in some way or other, and that the various articles about her might also possess a like power. She was particularly careful in regard to avoiding children, and would not on any account allow a child to touch or even to approach her closely. When she went out into the street she carefully gathered her skirts together on passing any person, for fear that she might by mere contact be contaminated. She spent hours every day in minutely examining and cleansing her combs and brushes, and was even then not satisfied that they were thoroughly purified.

As to her hands, she washed them, as her mother informed me she had ascertained by actual count, over two hundred times a day. She could touch nothing without feeling irresistibly impelled to scrub them with soap and water.

Gradually the idea of lice had been lost sight of, and for several months previously to her coming to me, the fear of pollution had had a much more extended source. She could not define with any exactness what the *materies pollutionis* was, though she imagined it to be something that was capable of doing her bodily injury in some subtle manner by being absorbed into her system through her hands or other parts.

Some little time before coming under my observatton she had extended her fear of contamination to the soap with which she felt compelled to wash her hands, and hence she was obliged to wash them again in pure water in order to remove all traces of the soap. Then, as the towel with which she wiped them dry had been washed with soap, she rinsed her hands in water, and allowed them to dry without the aid of a towel.

In removing her clothes at night preparatory to going to bed, she carefully avoids touching them with her hands, because then she would not have sufficient opportunity for washing. She, therefore, has some one else to loosen the fastenings, and then allows her garments to drop on the floor, where she leaves them. Nothing would persuade her to touch any of her under-clothing after it has been worn till it has been washed.

A great source of anxiety with her is the fact that her clothes are washed in the laundry with the clothing of other people, but she sees no practicable way of escape from this circumstance. It, none the less, however, makes her very unhappy.

When not washing her hands or examining her combs and brushes, she spends nearly all the rest of the day in carefully inspecting every article of furniture, and dusting it many times.

Thus her whole life is one continued round of trouble, anxiety and fear. Her whole character and disposition have changed. She is suspicious of every person and everything.

She is subject to insomnia, frequent headaches and loss of appetite. There are noises in her ears, flashes of light before her eyes, and an utter impossibility of concentrating the attention upon any other subject than the one which has obtained so complete a mastery over her. Her menstruation is scanty and somewhat painful, though regular in other respects.

Ophthalmoscopic examination showed the retinal vessels to be increased in size, and the choroid to be of a deeper hue than is ordinarily met with.

Upon conversing with this young lady I had no difficulty in getting her to admit the absurdity of her ideas. She stated that whenever she reflected upon the subject she was convinced of their erroneous character, but that nevertheless she could not avoid acting as she did, for as soon as she was exposed to any possible source of contamination, the ideas returned in full force. It was only when she had, as she thought, done her best to cleanse her hands, that she doubted the correctness of her notions which had so thoroughly become a part of her mentality.

Mysophobia is certainly closely allied with various hypochondriacal and hysterical forms of disease. Perhaps the nearest analogue is the sexual hypochondriasis or spermatophobia, of which all of us have seen many cases, and in which the miserable subject is continually haunted with the idea of impotence, softening of the brain, paralysis, etc., merely because he has an occasional nocturnal emission, or mistakes the escape of a little urethral mucus for the passage of semen. I have never, however, seen a case in a male, and though probably there are such, I am sure it is much more common with women than with men.

In several of my cases the affection had lasted two or three years before coming under treatment, and yet there did not appear to be any tendency to development into a more advanced type of mental derangement. The disorder appears to reach its full development in the course of a few months.

At no time is the intelligence of the patient weakened; even when the affection is in its height, or during the existence of a severe exacerbation, she is able to recognize the absurdity of the ideas which overpower her, and to resolve that she will endeavor to combat them; and although not successful in this contest, she nevertheless returns to the struggle day after day.

I have treated all my cases upon one general plan, and this has been so successful that I see no occasion for departing from it. As there is usually a tendency to constipation, I administer every alternate night a pill composed of 0.2 gram of podophyllin and 0.20 gram each of extract of aloes and inspissated ox-gall. Indeed, even were there no constipation I would use this pill, with the object of acting thoroughly on the liver and intestines. The intention is not to violently purge the patient, however, and therefore if it acts too powerfully, one of less quantity should be administered.

In addition I give the bromide of sodium or potassium or calcium, to such an extent as to bring the patient quickly and completely under its influence, and if there is a decided tendency to melancholia I give opium in combination.

It is not long before amelioration begins; the patient's mental strength improves day by day, and she is better able to contend with the ridiculous notions which govern her. The periods during which she is entirely convinced of her errors become longer and more frequent, and the false ideas themselves are less vivid. In the course of three or four months at the utmost, the patient is, according to my experience, free from mental aberration, though greatly reduced in strength. This, however, is not a matter of much consequence. The stoppage of the medication, and the administration of small doses of strychnia, iron and quinine, with cod-liver oil and a full, generous diet, will complete the cure.

All the cases under my care recovered, with the exception of the two now under treatment, and the result with them, as I judge by the steady improvement they have undergone, is merely a matter of time.

**Hydrastis Canadensis.**—Many of the peculiar virtues of hydrastis are probably due to the alkaloid berberine, which is contained in it in the proportion of about four per cent. In fact the so called hydrastin of the eclectics is really the muriate of berberine; while genuine hydrastin is the active principle of the plant, barring berberine, and is distinguished for the resemblance of its action both to quinine and pulsatilla.

In large doses it produces noises and a sensation of rushing in the ears, like those caused by quinine; and it is declared by Bartholow to rank next to quinine in the cure of intermittents, and by others to exceed quinine when there is that obstinate and obstructive complication of gastric and portal disturbance which renders some intermittents so intractable.

It will often cure chronic gastric catarrh and remove that distressing headache which frequently accompanies this disease.

Bartholow says:

"It is one of the best remedies for the stomach-catarrh of chronic alcoholism, and probably the best substitute, when given in full doses, for alcoholic stimulants when their use is sought to be abandoned."

Catarrh of the duodenum is also relieved by it, especially when accompanied by catarrh of the gall ducts and jaundice; and also catarrh of the cystic duct, with inspissation of the bile, and a tendency to gall stones.

In constipation from deficient secretion when the stools are dry and hard it may be depended upon, especially when combined with a little aloin; but torpor of the muscular coat of the intestines is not relieved by it, and requires the addition of ergot, nux vomica or physostigma.

Like pulsatilla it has been used in many other catarrhal affections, such as of the eyes, nose, ears. In follicular pharyngitis and chronic coryza, in chronic catarrh of the intestines and bladder, in chronic gonorrhœa and gleet, excellent effects have been noticed by Bartholow; who also, of course, declares it to be a most efficacious remedy in uterine and vaginal leucorrhœa, and in ulcerations and erosions of the os. It is also recommended in fissure of the anus, ulceration and hemorrhage from the rectal mucous membrane, although hamamelis is preferable; also, in unhealthy and sloughy sores, and old ulcers of the legs; even in syphilitic affections of the mouth, throat and nares, chancroids, and some other unhealthy growths. It is said to prevent septic decompositions in wounds and cavities communicating with the external air, and to be only second in efficiency to quinine and salicylic acid.

It is recommended in those glandular swellings which arise from absorption from diseased mucous membranes; while some fanciful authors think that conium is best adapted for those glandular affections which ensue from absorption from the diseases of the skin and other parts.—*The Physician.*

**Wind Spasm.**—Dr. Patterson reports to N. Y. Medical Record, that in the fall of 1877, while attending a lady for a slight indisposition, she informed him that her daughter was troubled with frequent eructations of wind, and wished to know if anything could be done to relieve her.

Upon further inquiry, says the Doctor, I found that she had been troubled with these spells, as she called them, for over six years, and that lately they had become so frequent and severe as to confine her to the house, not daring to go into society for fear they would come on, as they usually did, upon the least excitement. Her health was breaking down, she was losing flesh, had no appetite, and she feared that, unless a change could be brought about, some local disease might manifest itself, and she feared the result.

The young lady, on being questioned, expressed herself as follows (I use her own words, as far as I remember them):

"When these spells are coming on, I feel a sense of fullness which begins just below the pit of my stomach, and gradually goes all over me, even to the tip-end of my fingers and toes, until I feel literally as though I should burst, and I can get no relief, either from loosing my clothing or change of position, until at last I commence to belch up large quantities of gas or wind, which will continue to come up sometimes for an hour or more, and which always leaves me in a weak and exhausted condition."

She also informed me that it made no difference as to the kind or quantity of food she ate, and that they would come on sometimes just before meals, at other times just after, then again not for some hours after eating, and that they would sometimes occur during the night.

Having been under the care of several physicians, who had treated her for what they thought to be one of the complicated forms of dyspepsia, I thought myself that it might be one of the severer forms of the nervous type, and therefore treated her with the more common remedies, finally trying almost everything in the pharmacopeia that was suggested for such cases, but with no better success than my predecessors. At last, from my reading and observation of the case, I was led to try the sulphite of sodium, which I used as follows:

| | | |
|---|---|---|
| ℞ | Sodæ sulphis | ʒ ix. |
| | Aq. menth. pip | ℥ iv. |

S. One teaspoonful in half a wineglass of water, after eating.

This my patient took faithfully for two months. During the first two weeks she had four attacks, and there seemed to be no improvement; but after that time she made a steady and gradual improvement, the number and severity of the spasms diminished, she gained rapidly in health and spirits, and since the end of the eight weeks, although for a time she continued to take the medicine every other day and in smaller doses, she has been entirely free from them.

**Wickersheimer's Preservative Fluid.**—The composition of this fluid is as follows:

| | | | |
|---|---|---|---|
| ℞ | Alum | 100 | grammes. |
| | Common salt | 25 | " |
| | Saltpeter | 12 | " |
| | Potash | 60 | " |
| | Arsenious acid | 10 | " |

To be dissolved in 3000 gr. boiling water. On cooling, the liquid is to be filtered. To every two and a half litres, supposing a large quantity to be prepared at once, a litre of glycerine and 250 ccm. of methylic alcohol are to be added. Herr Wickersheimer states that the bodies of animals or men preserved with this fluid retain their form, color and pliability completely. After several years the muscles look as fresh on section as if they belonged to a recent corpse. For embalming purposes the body is first injected with the fluid, in the proportion of one litre and a half for a child of two years, and of five litres for an adult. It then is immersed in a bath of the fluid for several days, after which it is rubbed dry, swathed in bandages wetted with the fluid, and preserved in an air-tight case. For bodies which are to be dissected the injection alone suffices.

Small vertebrates and invertebrates can be kept simply immersed in the fluid, or if wanted in the dry state may be in it six to twelve days, and then be taken out and dried in the open air. Hollow organs, such as the lungs and intestinal tract, are best injected with it before immersion.

The process seems to have the recommendation of simplicity and cheapness, as well as that of its preserving the natural color and the pliability of the objects treated by it.—*Med. Times.*

**Death from Chloroform.**—Bardleben had witnessed more than 30,000 cases of chloroform narcosis up to 1876, without a death; in this year four fatal cases happened in his clinic, which has led him to employ in future only chloral-chloroform. The first case was that of a boy, aged 12, suffering from white-swelling of the knee, with acute angular contraction. He presented distinct evidences of scrofula and rachitis. Repeated auscultation and percussion excluded any morbid alterations of the thoracic organs. All precautions were taken; the stomach was empty, the clothes loose and horizontal posture secured. The anæsthetic was administered very slowly and with free admixture of atmospheric air. The operation contemplated (stretching of the crooked joint) was carried out without violence, loss of blood, or external wound. Suddenly the heart ceased, respiratory movements continuing quietly and without interference; a few minutes later the latter ceased also, and life could not be restored, although the most energetic efforts were made.

For similar cases, in which death evidently results from primary paralysis of the heart and not from suffocation, Bardleben recommends the subcutaneous use of sulph. strychnia, as proposed by Liebreich; in this case, the measure was resorted to, but too late to be of any avail.

Post-mortem revealed fluidity and dark color of the blood. The sinuses of the encephalon, the great veins of the diameter, and the heart cavities (of which the left ventricle was alone contracted) were full of blood. The heart structure appeared perfectly normal. At the apex of the left lung there was a small shrunken cheesy deposit, the size of a bean; the entire right lung was adherent by old and firm adhesions, was somewhat paler than the left, but completely pervious to air. The bronchial glands were as large as walnuts and contained cretaceous deposits.—*Maryland Med. Journal.*

**Citrate of Caffeine in Asthma.**—Dr. Throwgood, in Lancet, says:

In two cases recently I have observed excellent effects follow on the employment of the citrate of caffeine. One patient was an eminent medical practitioner in a large town in the North. He had suffered most severely from paroxysmal asthma, and the utter failure of a list of approved inhalations and medicines (far too long to be here enumerated), was most distressing. Four grains of citrate of caffeine produced an undue degree of wakefulness, but one grain taken regularly at bed-time, had a most happy effect indeed. So far as we can at present judge, it appears to have been really curative of the asthma. The last report says: "*Pari passu* with the asthma my cough and expectoration have gone, and I now have next to none of either."

In another case, a highly informed and observant patient of Dr. Kingsford's, who had found much benefit from the inhalation of iodide of ethyl for an asthma and bronchitis of twenty years' standing, tried the citrate of caffeine in two-grain doses every afternoon for a fortnight without any marked result. One day, however, being sadly worn out by a protracted attack of bronchial spasm which had lasted for eight hours, this patient took four grains of citrate of caffeine in coffee, with the effect of obtaining immediate relief to the spasm followed by three hours' quiet sleep in his chair. The citrate of caffeine appears to allay the abnormal excitability of the nerve-centers, and then repose ensues as a natural result. I have seen similar calming effect from the use of nux vomica in bad emphysematous asthma, with failing pulmonary innervation, and this soothing and soporific action of remedies reputed nerve-tonics and stimulants in cases of great exhaustion of the nerve-centers is an interesting therapeutical fact, ably set forth by Dr. Milner Fothergill in his Fothergillian prize essay.

**Hemorrhage in Abortion.**—Dr. Griswold, President of Hartford Medical Association, says, in Louisville Medical Journal:

For the last twenty years my reliance has been on a junk of alum in the vagina. If this is not at hand I take the next best thing that is; but a junk of alum is a part of the contents of my medicine-box. It is of the size of a large hen's egg, ovoid in shape, and generally left a little ragged, though without sharp points. Around the middle is cut a groove, about which is tied a bit of strong but not large twine, leaving the ends so that they can hang out of the vagina. No preparation is necessary nor any exposure of the person needed. The egg is introduced endway, turned half round so as to bring the long diameter across the vagina, and pushed downward and then upward against the os. In some cases, especially if the canal is large, I back the egg with sufficient packing to secure its retention in position. If the vagina be small and close, there may be no need at all of the supplementary support.

This treatment is easy, speedy and effectual against further hemorrhage. It has never failed me, and I leave a patient with the feeling that she is safe for the next twelve or fifteen hours, so far as danger from further bleeding is concerned. And I may add that I have never had any unfavorable effects follow its use in any one of the scores of cases in which it has been employed—no fevers, no septicemia, no deaths, no anything untoward—and I have never had occasion to use it the second time in any one case. It can be removed, when desirable either by traction on the cord or by the introduction of the fingers, the coagulated blood fished out, the vagina syringed, and the case further treated as circumstances may require.

Perhaps this is nothing new; but as it is something I have not seen mention made of in any of the standard works that have come under my observation, nor in special papers, nor have ever heard of in the lectures of the schools, I venture to submit it to your columns, and through them to professional notice.

**How to Postpone the Use of Spectacles.**—Dr. W. Cheatham, in the Louisville Medical News, says:

Till lately I had advised the use of spectacles the instant their want is felt, but now we have in sulphate of eserine a remedy (and a safe one, I believe), by which the wearing of glasses can be put off for several years. In presbyopia we have loss of distinct near vision, caused partly by the loss of power in what is known as the ciliary muscle. Eserine is a stimulant to this muscle, producing a contraction, and in that way assists in accommodation.

From my results so far I believe that spectacles may be dispensed with for several years after their want is first felt. I usually order:

℞ Eserine sulphat........................................ gr. j.
Aquæ dist............................................ ℥j.

One drop to be put into each eye at bedtime. On account of the artificial myopia produced I order it to be put in at bedtime. It may be dropped in at any time, as the myosis soon passes away.

Besides its employment in glaucoma and other inflammations of the eye, and in presbyopia, I have found it of great use in asthenopic (weak) eyes, depending upon over-sightedness and weakness of accommodation, the latter the result of either overwork, general debility, diphtheria, etc.

Spectacles in presbyopia (the loss of near vision from age) always give ease; but there is a certain discomfort from the use of glasses, besides many objections brought forward by patients, all of which, as a usual thing, can be referred to pride. This pride we should humor as much as possible. If by means of the eserine we can give them as great comfort and preserve their eyes as well as by means of spectacles, I think it proper that we should do so.—*American Med. Journal.*

**Treatment of Hepatic Calculi.**—Dr. T. H. Buckler, N. Y. Medical Journal, in referring to Dr. T. J. Thomas's enumeration of the operation of cutting into the gall-bladder as one of the recent surgical triumphs, asserts that such procedure is unwarrantable. Cholesteric gall-stones can always be dissolved away by large doses of chloroform, especially if combined with succinate of iron. The latter agent also may alone accomplish the desired solution and effect a cure. In Dr. Buckler's last three cases, treated successfully, he gave ten drops of chloroform every four hours, and a teaspoonful of Steward's hydrated succinate of the peroxide of iron half an hour after each meal. He has sometimes given a teaspoonful of chloroform every six hours, without causing any bad symptoms and with the result of a cure within a week. The succinate of iron contains, according to Dr. Buckler, more nascent appropriate oxygen than any other known therapeutic agent, and is one of the best ferruginous preparations, apart from its solvent powers on gall-stones. It is better than nitric acid in affections of the liver. Chloroform, we are told, on being swallowed, passes into the acini of the liver, then into the bile of the gall-bladder, where it dissolves the gall stone with the inexorable certainty of mathematics. Dr. Buckler's experience with ether, and with the various mineral waters, has led him to consider them of no value in this trouble.—*Canada Lancet.*

[A teaspoonful of chloroform is too large a dose except when the pain is excruciating. We have known it to produce delirium and decided intoxication on more than one occasion.—ED.]

**A Simple Apparatus for the Treatment of Fractures of the Clavicle.**—Dr. C. A. Dugas, in the N. O. Med. and Surg. Journal for January, describes his method of treating fractures of the clavicle. He discards all axillary pads as inefficient and injurious. To meet the usual indications in these fractures he prepares and applies an apparatus as follows :

A square yard of unbleached shirting is cut diagonally so as to form two triangular pieces. To each of the acute angles of one of these pieces a three-inch bandage, four yards long, is sewed. This completes the apparatus. The displacement is then reduced by carrying the shoulder upward, backward and outward. Then the middle of the long side of the triangle is applied beneath the elbow, leaving a margin of four inches behind, the right angle being directed toward the fingers. One of the acute angles with its bandage is now carried between the arm and chest, up to the fractured clavicle, around the back of the neck, over the sound shoulder in front and beneath the axilla, and finally around the arm just above the elbow. The other end of the strip is then carried up, in front of the forearm, to the sound shoulder, behind and beneath the axilla, and around the chest and arm, so as to meet its fellow to be tied to it. Finally, the margin left projecting behind the elbow should be elevated, doubled and stitched, so as to prevent the elbow from sliding out. The strips encircling the arm should also be stitched to prevent displacement.

This bandage is said to be a very comfortable one, easily applied, and efficient.—*Med. Record.*

**Coptis Trifoliata—Gold Thread.**—The ranunculus, or crow-foot tribe of plants, although generally powerful, includes some very mild articles like the coptis trifoliata, or gold-thread, which has few special virtues beyond those of the simple bitters to recommend it; but in case of need may be substituted for them, because the bright yellow creeping root from which its popular name is derived is intensely bitter. In New England it is commonly used as a wash for aphthous sore mouth, although there is little or no evidence of its peculiar virtues in this complaint. In short, it is a simple bitter tonic, with some of the anti-periodic virtues of berberine.

It is not an astringent, for no tannin has been found in it; but, like several other plants characterized by bitterness and a yellow color, it contains the alkaloid berberina, than which few, if any, of the known alkaloids are so widely diffused in the vegetable kingdom.

Berberina is especially abundant in the hydrastis canadensis, calumba, and other plants, and there is little doubt from its extensive diffusion in plants used in medicine that it is possessed of valuable remedial properties.

One to ten grains of the muriate of berberine may be used per dose.
—*The Physician.*

**Counter-Irritation in Febrile Phthisis.**—In Compendium, Dr. L. M. James gives his opinion on the value of counter-irritation. I think, he says, that remedy has, in our own country, gone into comparative but undeserved disuse in all the forms of consumption. I notice frequently the best of results from its use.

The best forms of counter-irritation in chronic phthisis, ordinarily, I think, are a succession of small blisters, or the use of the tincture or compound tincture of iodine, repeated sufficiently often to produce decided irritation, but not to torture the patient. The inhalation of the fumes of the iodine, when applied to the front of the chest, possibly has some good effect.

When pneumonia, pleurisy, or active congestions of the pulmonary structure supervene in the progress of chronic phthisis, I regard counter-irritation as the best remedy available, and a blister the best form of counter-irritation, used of a size to produce the requisite impression. When attended with high febrile disturbances, antipyretic doses of quinine are suitable.

**Treatment of Chest Diseases by Petroleum.**—Dr. Moubre, writing to the *Gazette des Hopitaux*, gives his experience of petroleum capsules in simple and chronic bronchitis. This balsamic had been brought before the Therapeutic Society by Dr. Blache a year ago, at the instigation of a Paris chemist, who named it Gabian oil, in order to prevent public prejudice. Each capsule contains twenty-five centigrammes of pure petroleum, the ordinary oil not being used, as it has to be distilled in contact with sulphuric acid to render it fit for lighting purposes. At the Hospital Beaujon, where these capsules have been freely ordered for chronic bronchitis, a rapid diminution of the secretion and fits of coughing were observed. In tuberculosis this medicine gave encouraging results.—*Med. and Surg. Reporter.*

**Glycerine in Phthisis.**—Glycerine, says Dr. L. M. James, in Compendium, has been frequently used in phthisis. He has used it himself with good results. Physiological experiments show that it promotes the appetite, increases flesh, prevents tissue-waste, as revealed by the diminution of the amount of urea excreted, and becomes directly oxidized—all of them important ends in the treatment of phthisis. It is also found very useful in controlling cough. His own experience with it has been chiefly in combination with alcoholics.

**Phosphorus and its Combinations.**—My own experience with phosphorus has been limited to the use of the rypophosphites. With them uncombined, I have not derived very satisfactory results; but in combination with cod liver oil, they have seemed to exert a good influence.—Dr. L. M. James *in Compendium.*

